# Sodium phenylacetate enhances the inhibitory effect of dextran derivative on breast cancer cell growth in vitro and in nude mice

**DOI:** 10.1054/bjoc.2001.1993

**Published:** 2001-09

**Authors:** M Di Benedetto, Y Kourbali, A Starzec, R Vassy, J Jozefonvicz, G Perret, M Crepin, M Kraemer

**Affiliations:** UPRES 2360, Equipe d'Oncologie cellulaire et moléculaire Université Paris 13, 74 Rue Marcel Cachin, Bobigny cedex, 93017, France; UPRES 2360, Equipe de Pharmacologie, Bobigny, 93017; LRM, CNRS, URM 502, Institut Galilée, Université Paris 13, Avenue JB Clément, Villetaneuse, 93340, France

**Keywords:** sodium phenylacetate (NaPa), carboxymethyl benzylamide dextran derivative (CMDB), synergism, MCF-7ras, breast cancer

## Abstract

Sodium phenylacetate (NaPa) and carboxymethyl benzylamide dextran derivative (CMDB_LS4_) are able to inhibit growth of breast tumour cells. In this study, we explored whether the combination of NaPa and CMDB_LS4_ may enhance their respective inhibitory effects on the MCF-7ras cell growth in vitro and in vivo. NaPa inhibited MCF-7ras cell proliferation by reducing the DNA replication concomitantly with a recruitment of cells in G0/G1 phase and by inducing apoptosis in a dose- and time-dependent manner. The addition of CMDB_LS4_ potentiated the NaPa antiproliferative effect in the manner dependent on the ratio of CMDB_LS4_ and NaPa concentrations. In nude mice, CMDB_LS4_ (150 mg kg^−1^) or NaPa (40 mg kg^−1^) administrated twice a week, for 7 weeks inhibited MCF-7ras xenograft growth by 40% and 60%, respectively. The treatment by both, CMDB_LS4_ and NaPa, decreased tumour growth by 83% without any toxicity. To better understand the mechanism of NaPa and CMDB_LS4_ action we assessed their effect on mitogenic activity of MCF-7ras conditioned medium (CM) on BALBC/3T3 fibroblasts. CMDB_LS4_ added to the CM, inhibited its mitogenic activity whereas NaPa had an anti-mitogenic effect when CM was prepared from MCF-7ras cells pretreated with NaPa. Thus, the antiproliferative effects of NaPa and CMDB_LS4_ involve 2 different mechanisms explaining, at least in part, the possible synergism between them. Overall, this study points to the potential use of a combination of dextran derivatives with NaPa to inhibit the breast tumour growth. © 2001 Cancer Research Campaignhttp://www.bjcancer.com


*British Journal of Cancer* (2001) **85**(6), 917–923
© 2001 Cancer Research Campaign
doi: 10.1054/ bjoc.2001.1993, available online at http://www.idealibrary.com on http://www.bjcancer.com

### Sodium phenylacetate enhances the inhibitory effect of dextran derivative on breast cancer cell growth in vitro and in nude mice

**M Di Benedetto[1], Y Kourbali[1], A Starzec[2], R Vassy[2], J Jozefonvicz[3], G Perret[2], M Crepin[1] and M Kraemer[1]**

[1]UPRES 2360, Equipe d'Oncologie cellulaire et moléculaire Université Paris 13, 74 Rue Marcel Cachin, 93017 Bobigny cedex, France; [2]UPRES 2360, Equipe de Pharmacologie, 93017 Bobigny; [3]LRM, CNRS, URM 502, Institut Galilée, Université Paris 13 Avenue JB Clément 93340 Villetaneuse, France

**Summary** Sodium phenylacetate (NaPa) and carboxymethyl benzylamide dextran derivative ($CMDB_{LS4}$) are able to inhibit growth of breast tumour cells. In this study, we explored whether the combination of NaPa and $CMDB_{LS4}$ may enhance their respective inhibitory effects on the MCF-7ras cell growth in vitro and in vivo. NaPa inhibited MCF-7ras cell proliferation by reducing the DNA replication concomitantly with a recruitment of cells in G0/G1 phase and by inducing apoptosis in a dose- and time-dependent manner. The addition of $CMDB_{LS4}$ potentiated the NaPa antiproliferative effect in the manner dependent on the ratio of $CMDB_{LS4}$ and NaPa concentrations. In nude mice, $CMDB_{LS4}$ (150 mg $kg^{-1}$) or NaPa (40 mg $kg^{-1}$) administrated twice a week, for 7 weeks inhibited MCF-7ras xenograft growth by 40% and 60%, respectively. The treatment by both, $CMDB_{LS4}$ and NaPa, decreased tumour growth by 83% without any toxicity. To better understand the mechanism of NaPa and $CMDB_{LS4}$ action we assessed their effect on mitogenic activity of MCF-7ras conditioned medium (CM) on BALBC/3T3 fibroblasts. $CMDB_{LS4}$ added to the CM, inhibited its mitogenic activity whereas NaPa had an anti-mitogenic effect when CM was prepared from MCF-7ras cells pretreated with NaPa. Thus, the antiproliferative effects of NaPa and $CMDB_{LS4}$ involve 2 different mechanisms explaining, at least in part, the possible synergism between them. Overall, this study points to the potential use of a combination of dextran derivatives with NaPa to inhibit the breast tumour growth. © 2001 Cancer Research Campaign http://www.bjcancer.com

**Keywords**: sodium phenylacetate (NaPa); carboxymethyl benzylamide dextran derivative (CMDB); synergism; MCF-7ras; breast cancer

We recently showed that carboxymethyl benzylamide dextran derivative (in particular CMDB7) inhibits the breast cancer cell proliferation in vitro and in nude mice (Bagheri-Yarmand et al, 1992, 1997, 1998a, b, 1999). This effect in vitro is associated with a decrease in the S-phase cell population and with an accumulation of cells in G1 phase of cell cycle (Bagheri-Yarmand et al, 1992). CMDB7 disrupts the mitogenic effect of growth factors by preventing their binding to specific receptors as reported for Fibroblast Growth Factor-2 and -4 (FGF2, FGF4, Bagheri-Yarmand et al, 1998a), Platelet-Derived Growth Factor-BB and Transforming Growth Factor-β1 (PDGF-BB, TGFB Bagheri-Yarmand et al, 1998b). In vivo, CMDB7 treatment reduces the growth of MCF-7ras (Bagheri-Yarmand et al, 1998b) and FGF4-transfected HBL100 xenografts and decreases the tumour angiogenesis (Bagheri-Yarmand et al, 1998a).

Sodium phenylacetate (NaPa), a physiological metabolite of phenylalanine, is normally found in human plasma at micromolar concentrations. At higher concentrations, NaPa was reported to induce the cytostasis and the reversion of malignant phenotype of different cancer cells in vitro (Samid et al, 1993, 1994, 1997, 2000; Adam et al, 1995). Furthermore, NaPa was described to modulate the synthesis and/or the release of some growth factors (Ferrandina et al, 1997; Thibout et al, 1998) and to increase, in synergistic manner, the effect of some molecules affecting the growth factor pathways (Samid et al, 1993; Prasanna et al, 1996). For example, NaPa potentiated the antitumour activity of tamoxifene by increasing apoptosis induction in breast cancer xenografts in nude mice. Finally, NaPa had been used in phase I and II clinical trials on patients with malignant tumours (Thibault et al, 1994; Chang et al, 1999).

In this study, we investigated in vitro and in vivo the efficacy of combined treatment with NaPa and an industrial dextran derivative LS4 (Sterilyo Laboratories) whose composition is similar to CMDB7 one, on breast cancer cell growth. We have used the MCF-7ras cell line obtained by transfection of MCF-7 cells, isolated from pleural metastasis of breast adenocarcinoma, with v-Ha-ras oncogene. The MCF-7ras cells secrete high quantities of TGFα, TGFβ, epithelial growth factor (EGF) and insulin growth factor (IGF) (Albini et al, 1986). This cell line represents an oestrogen-independent cellular model corresponding to some malignant breast tumours (Spandidos and Agnantis, 1984) and it no requires the oestrogen supplementation to induce a high incidence of tumours in nude mice (Sommers et al, 1990). The analysis of $CMDB_{LS4}$-NaPa combination effect was performed by the isobole method. Then, we explored the mechanism of action of $CMDB_{LS4}$-NaPa combination.

### MATERIALS AND METHODS

### Compound preparation

NaPa was provided by Seratec (Paris, France). Industrial water-soluble dextran derivative CMDB named $CMDB_{LS4}$, was synthetized by Sterilyo Laboratories (Levallois-Perret, France)

*Received 8 January 2001*
*Revised 14 May 2001*
*Accepted 12 June 2001*

*Correspondence to:* M Di Benedetto with some modifications of the procedure described by Mauzac and Jozefonvicz (1984) and by Chaubet et al (1995). In brief, $CMDB_{LS4}$ was prepared by simultaneous carboxymethylation and benzylamidation of dextran T40 in one step. After purification by ultrafiltration and lyophilization, the chemical composition (or degree of substitution, ds) of $CMDB_{LS4}$ was determined by acidimetric titration and elementary analysis of nitrogen. The dextran, carboxymethyl (CM) and benzylamide (B) was 0, 0.67 and 0.33, respectively. The average molecular weight, calculated considering CMDB subunits was $PM_{T40} + ds_{CM} \times PM_{CM} + ds_{B} \times PM_{B} =$ 264.1 g $mol^{-1}$.

### Cell culture

The human breast cancer MCF-7ras cells, derived from the pleural effusion MCF-7 cells transfected with v-Ha-ras, were kindly provided by Dr C Sommers (Georgetown University, Washington, DC, USA). BALB/c3T3 fibroblasts were purchased from American Tissue Culture Collection (Rockeville, MA, USA). All cells were routinely grown in DMEM supplemented with L-glutamine 2 mM, penicillin 50 IU $ml^{-1}$, streptomycin 50 μg $ml^{-1}$, and fetal calf serum 10% (FCS) (Life Technologies, Inc, Gaithersburg, MD, USA) at 37˚C in a 5% $CO_2$ humidified atmosphere.

### Growth inhibition experiments

MCF-7ras cell growth was assessed using the MTT-microculture tetrazolium assay (Mosmann, 1983). As previously described, CMDB7 antiproliferative effect is FCS concentration-dependent with an optimum observed in the presence of 1 to 2% of FCS (Bagheri-Yarmand et al, 1992). Briefly, the cells ($4 \times 10^3$) were incubated in 2% FCS/DMEM for 24 h and then treated with NaPa or/and $CMDB_{LS4}$ at different concentrations for 72 h. Then, the cells were washed with PBS and incubated with 0.1 ml of MTT 2 mg $ml^{-1}$. Complementary assay of cell cytotoxicity was performed by trypan blue exclusion test.

For analysis of DNA synthesis, the bromodeoxyuridine (BrdU) (Boehringer Kit, Mannheim, Germany) labelling was used. After incubation of MCF-7ras cells with NaPa at concentrations 1–40 mM, the BrdU 10 μM was added for 6 h and its uptake was measured by monoclonal antibody from mouse–mouse hybrid cells conjugated with peroxidase (1/100). After 3 washes with PBS, the substrate reaction with tetramethyl-benzidine was stopped with 25 μl of $H_2SO_4$, then the absorbance was determined at 405 nm wavelength using an microplate reader (Biorad, Marnes-La-Coquette, France).

The drug interaction between NaPa and $CMDB_{LS4}$ was evaluated by comparison of the dose–response curves of MTT assay for single agents and combined treatment. The characteristics of the combined treatment were then analysed by the isobole method (Berenbaum, 1981) analysing the equation Ac/Ae + Bc/Be = D, where Ac and Bc correspond to concentrations of drugs A and B used in the combined treatment, and Ae and Be are the concentrations of drugs able to produce the same magnitude of effect if used individually. If D (combination index) < 1, the effect of combination is synergistic, when D = 1 its effect is additive and in the case of D > 1 the combined action is antagonistic. Each experiment was performed in triplicate and the statistical significance ($P$) of the difference between D obtained and D = 1 was calculated by the Student's $t$-test.

### Preparation of conditioned medium (CM)

MCF-7ras cells ($1 \times 10^6$) were treated with or without NaPa 20 mM for 48 h in DMEM supplemented with FCS 2% and washed twice with PBS. To obtain conditioned medium (CM) containing only the growth factors produced by MCF-7ras, the cells were incubated with serum-free DMEM for 24 h at 37˚C. This medium (CM) was then harvested and used directly.

### CM experiments

BALB/c3T3 fibroblasts ($5 \times 10^4$) were grown until preconfluence in DMEM supplemented with FCS 10%, washed twice with PBS and growth-arrested by serum starvation for 24 h. Then, 0.75 ml of the CM from MCF-7ras cells, treated or not with NaPa, was added to 0.25 ml of DMEM containing or not the $CMDB_{LS4}$ or NaPa. After 48 h, the proliferative effects of the CM was measured by counting the fibroblasts using a Coulter counter (Coultronics, Margency, France).

### Cell cycle analysis

MCF-7ras cells ($5 \times 10^4$ $ml^{-1}$) were plated in DMEM supplemented with FCS 2%. After 24 h of culture, NaPa was added for 72 h. Then, the cells were treated with BrdU (Pharmingen, San Diego, CA, USA) for 4 h. The incorporated BrdU was revealed using an anti-BrdU conjugated with fluorescein isothiocyanate (Boerhinger Mannheim, Germany). Then, the cells were stained with propidium iodide (PI) (Boerhinger) for 10 min and were analysed using a flow cytometer (Coulter Epics Laser, CA, USA).

### Evaluation of cell death

Trypan blue and propidium iodide (PI) enter into the cells during the ultimate stage of apoptosis or predominantly the first stage of necrosis when damage of the cell membrane has occurred. After the different treatment, the cells were harvested and the cell viability was determined by trypan blue exclusion. Other aliquot of the same sample cells were centrifuged and washed with annexin buffer (Boehringer). To reveal a phosphatidylserine translocation specific to apoptosis stage, the cells were treated with a FITC-labelled annexin V (Boerhinger) followed by PI (Boerhinger) and finally the cells were analysed using a flow cytometer.

### Xenografts in nude mice

MCF-7ras cells ($5 \times 10^6$) were inoculated s.c. near the right mammary fad pad of 3-week-old athymic nude mice (nu/nu) (Harlan laboratory, Gannat, France). The animals ($n$ = 40) were kept in a temperature-controlled room on a 12h:12h light–dark schedule with food and water ad libitum. This protocol resulted in the development of single s.c. palpable tumours 4 weeks after cell inoculation in 90% of the mice. Then, the animals were arbitrarily placed in control ($n$ = 10) and treated groups ($n$ = 10). NaPa (40 mg $kg^{-1}$), $CMDB_{LS4}$ (150 mg $kg^{-1}$) or $CMDB_{LS4}$/NaPa combination (150/40 mg $kg^{-1}$, respectively) were injected s.c. in 0.1 ml of NaCl 0.9% twice a week for 7 weeks. The control group received s.c. 0.1 ml of NaCl 0.9%. A tumour volume was calculated as previously described (Bagheri-Yarmand et al, 1998a).

 © 2001 Cancer Research Campaign

### Statistical analysis

Multiple statistical comparisons were performed using ANOVA in a multivariable linear model. Some statistical comparisons were conducted using the Mann–Whitney *t*-test. $P < 0.05$ was considered statistically significant.

### RESULTS

### Inhibition of MCF-7ras cell growth by NaPa

After 72 h exposure, NaPa induced a dose-dependent inhibition ($IC_{50}$ = 20 mM) of MCF-7ras cell growth (Figure 1A). This decline was associated with a similar inhibition of DNA synthesis (Figure 1A) as well as with a decrease in MCF-7ras cell number in S-phase and a major recruitment of cells in the G0/G1 phase (Figure 1B).

NaPa induced the cell death but only at high concentration (40 mM) (Figure 2A). At concentrations ranged from 5 to 30 mM NaPa did not induce apoptotic, necrotic or toxic effects as showed by the negative results of trypan blue exclusion test and annexin V labelling. In the presence of NaPa 40 mM, we observed 40% of cells double labelled with annexin V/PI. Moreover, this result was confirmed using trypan blue exclusion test. The apoptotic effect of NaPa 40 mM on MCF-7ras cells was time-dependent (Figures 2B and 3). No apoptotic or necrotic effects were observed after 24 h of treatment (data not shown). After 48 h of treatment, 2 separate annexin V positive cell populations were observed. The first one represented 10% of total cells and contained annexin V single stained cells being in an early apoptotic phase. The other one included 18% of annexin V/PI double stained cells or trypan blue-stained cells progressing in a later apoptotis or in the early stage of necrosis. After 72 h of treatment, only annexin V/PI double-stained cell population (40%) was found.

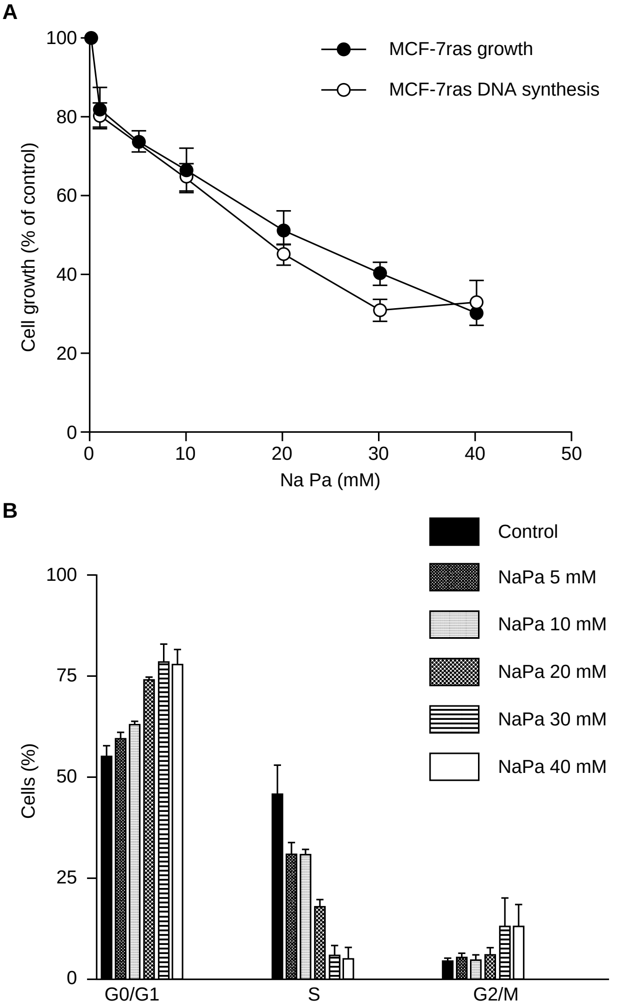

**Figure 1** Effect of NaPa on MCF-7ras proliferation (**A**) and cell cycle distribution (**B**). (**A**) Cells treated with NaPa for 72 h were assessed using MTT-assay as described in 'Materials and Methods'. Points represent the mean (± SD) of 3 independent experiments. (**B**) Distribution of cells treated with or without NaPa 5–40 mM in different phases of cell cycle. Each column represents a mean (± SD) of 3 independent experiments

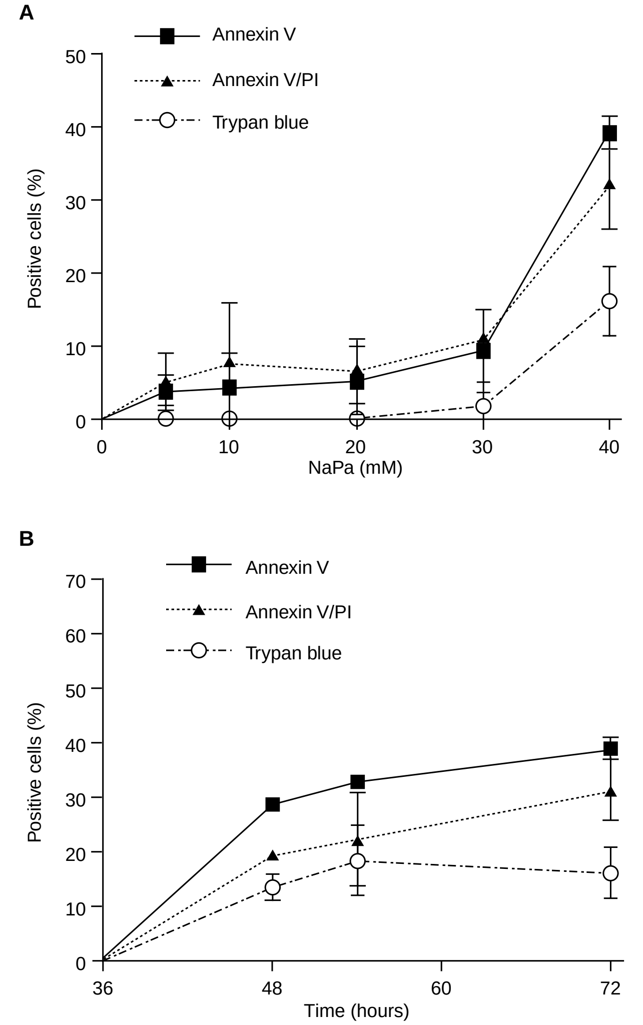

**Figure 2** Apoptosis of MCF-7ras induced by NaPa. (**A**) Apoptosis of MCF-7ras cells treated with NaPa at various concentrations for 72 h. (**B**) Apoptosis of MCF-7ras cells treated with NaPa 40 mM for various time. Each point represents the mean (± SD) of 3 independent experiments

### Improvement of MCF-7 ras cell growth inhibition by $CMDB_{LS4}$-NaPa combination

As shown in Figure 4, the antiproliferative effect of $CMDB_{LS4}$ on MCF-7ras cells was potentiated in the presence of NaPa 5 mM. Indeed, $CMDB_{LS4}$ 18 mM alone was not able to inhibit the MCF-7ras cell growth by more than 38% ($IC_{50}$ not reached), whereas in

**Table 1** Growth of MCF-7ras cells treated with different $CMDB_{LS4}$/NaPa combined ratio

| $CMDB_{LS4}$/NaPa ratio | 0.01 | 0.1 | 1 | 2 | 5 | 8 |
|---|---|---|---|---|---|---|
| Concentrations (mM) | C 0.05, N 5 | C 0.5, N 5 | C 5, N 5 | C 10, N 5 | C 18.5, N 3.7 | C 18, N 2.25 |
| $CMDB_{LS4}$ | 87.5 ± 0.8 | 82 ± 2 | 73 ± 1.7 | 69 ± 1 | 65 ± 2 | 65 ± 2 |
| NaPa | 74 ± 1.7 | 74 ± 1.7 | 74 ± 1.7 | 74 ± 1.7 | 78 ± 2 | 78 ± 0.8 |
| $CMDB_{LS4}$+NaPa | 75 ± 2.8 | 70 ± 0.5 | 57 ± 1.8* | 51 ± 0.8* | 44 ± 2.2* | 44 ± 2* |

MCF-7ras cells were treated with various ratios of $CMDB_{LS4}$/NaPa (0.01, 0.1, 1, 2, 5, 8). Cell growth was determined by MTT assay (see 'Materials and Methods'). Each value represents the mean of 3 independent experiments. C = Concentration of CMDBLS4 (mM); N = Concentration of NaPa (mM); **P* < 0.05 versus $CMDB_{LS4}$ or NaPa alone.

**Table 2** Effects of NaPa and $CMDB_{LS4}$ combination with a ratio = 5 on MCF-7ras cell proliferation

| [a]$CMDB_{LS4}$ ($A_c$; mM) | NaPa ($B_c$; mM) | % I | $CMDB_{LS4}$ ($A_e$; mM) | NaPa ($B_e$; mM) | D |
|---|---|---|---|---|---|
| 48 h | | | | | |
| 3.7 | 0.75 | 33 | 18.5 | 17 | 0.44[b] |
| 7.4 | 1.5 | 45.8 | >18.5 | 30 | 0.45[b] |
| 14.8 | 3 | 48.1 | >18.5 | 32.1 | 0.89 |
| 18.5 | 4 | 50 | >18.5 | 34 | 1.12 |
| 72 h | | | | | |
| 3.7 | 0.75 | 51.4 | >18.5 | 20.6 | 0.23[b] |
| 7.4 | 1.5 | 54.8 | >18.5 | 23 | 0.46[b] |
| 14.8 | 3 | 53.3 | >18.5 | 24 | 0.93 |
| 18.5 | 4 | 60.2 | >18.5 | 29 | 1.13 |

[a] $A_c$ and $B_c$, concentrations of agents A and B used in the combination treatment; $A_e$ and $B_e$, concentrations of agents A and B able to produce the same magnitude of effect if used individually. D combination index (see Materials and Methods). [b] P < 0.05. % of inhibition determined by MTT assay: [1–(absorbance of cells in medium containing agents/absorbance of cells in control culture medium)] × 100.

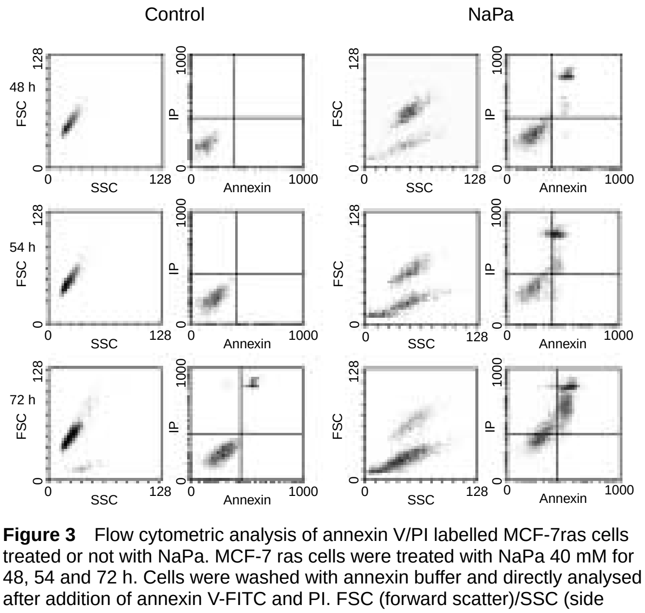

**Figure 3** Flow cytometric analysis of annexin V/PI labelled MCF-7ras cells treated or not with NaPa. MCF-7 ras cells were treated with NaPa 40 mM for 48, 54 and 72 h. Cells were washed with annexin buffer and directly analysed after addition of annexin V-FITC and PI. FSC (forward scatter)/SSC (side scatter) pattern are shown in column 1 for control and 3 for NaPa treatment and annexin V/PI staining profile are shown in column 2 (control) and 4 (NaPa)

the presence of NaPa 5 mM, $CMDB_{LS4}$ was more potent and reduced the cell growth with an $IC_{50}$ of 12 mM.

In order to better characterize the inhibition of MCF-7ras cell growth by $CMDB_{LS4}$-NaPa combination, we evaluated this inhibitory effect for various $CMDB_{LS4}$ and NaPa concentrations and for their different ratios ($CMDB_{LS4}$/NaPa) in a range from 0.01 to 8 (Table 1). The inhibition of MCF-7ras cell growth induced by $CMDB_{LS4}$–NaPa combination was compared to their single respective effect. Only for $CMDB_{LS4}$/NaPa ratios > 1 the inhibitory effect was potentiated.

For $CMDB_{LS4}$/NaPa ratio = 5, an additive inhibition (40–50%) was observed after 48 h and 72 h when higher drug concentrations were used (Table 2). A synergistic inhibitory effect was detected for lower concentrations of $CMDB_{LS4}$ and NaPa. In all cases of combined treatment, an increase in MCF-7ras cell growth inhibition was time-dependent. When used in combination, NaPa and $CMDB_{LS4}$ are efficient at much lower concentrations than when applied separately to induce the same inhibitory effect. The best combination seems to be 3.7 mM $CMDB_{LS4}$/0.75 mM NaPa. The different combined treatments were not cytotoxic as determined by a trypan blue exclusion test (data not shown).

### Effect of NaPa and $CMDB_{LS4}$ on MCF-7 ras CM mitogenic activity on fibroblast proliferation

Previous reports indicated that MCF-7ras cells secreted the growth factors mitogenic for BALBc/3T3 fibroblasts (Bagheri-Yarmand et al, 1998a). We investigated whether $CMDB_{LS4}$ or NaPa could influence the mitogenic activity of MCF-7ras cell conditionned medium (CM) (Figure 5). CM stimulated BALBc/3T3 fibroblast proliferation 2-fold after 48 h of treatment. The addition of $CMDB_{LS4}$ 18.5 mM abolished the MCF-7ras CM stimulation of

 *© 2001 Cancer Research Campaign*

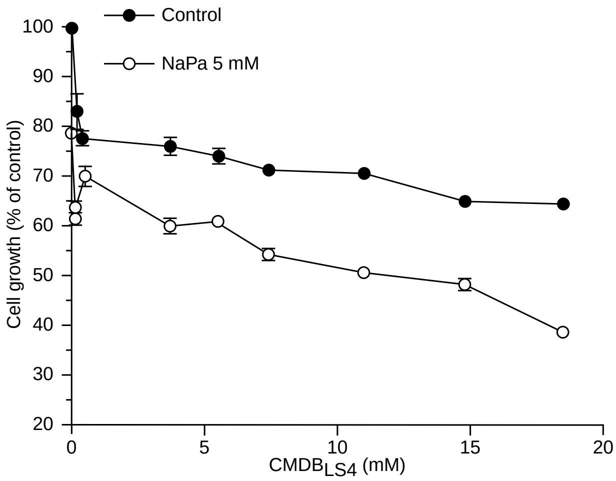

**Figure 4** Antiproliferative effect of $CMDB_{LS4}$ in the presence of NaPa on MCF-7ras cell proliferation. The cells were treated with NaPa 5 mM and increasing concentrations of $CMDB_{LS4}$ for 72 h. Each point represents the mean (± SD) of 3 independent experiments

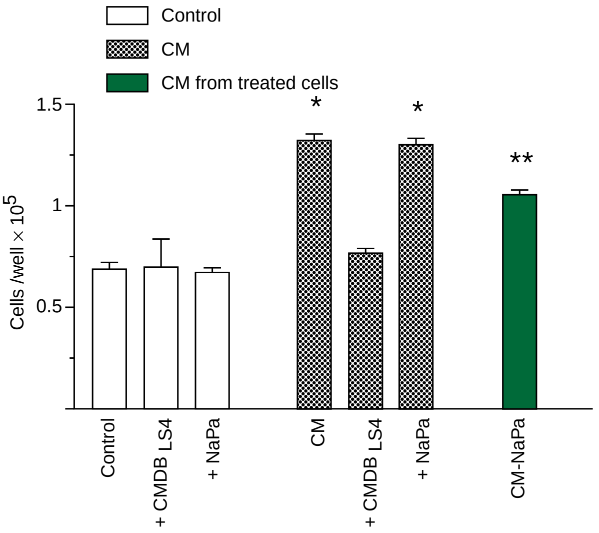

**Figure 5** The effects of $CMDB_{LS4}$ and NaPa on the mitogenic activity of MCF-7ras conditioned medium (CM). CM from MCF-7ras cells treated (CM-NaPa) or not (CM) with NaPa 20 mM was tested for its effect on BALBc/3T3 fibroblasts growth. Control represents the fibroblasts treated with serum-free medium (see 'Materials and Methods'). Each column represents the mean (± SD) of 3 independent experiments. $^*P < 0.05$ versus control, $^{**}P < 0.05$ versus control and CM

fibroblasts growth. In contrast, NaPa 20 mM addition did not affect this CM. However, the pre-treatment of MCF-7ras cells with NaPa 20 mM for 48 h made a CM less effective on fibroblast proliferation.

### Antitumour activity of $CMDB_{LS4}$-NaPa combination on MCF-7ras cell xenografted into nude mice

We focused our attention on the in vivo effect of $CMDB_{LS4}$-NaPa combination in a ratio of $CMDB_{LS4}$/NaPa > 1 (Figure 6). NaPa (40 mg $kg^{-1}$ twice a week) inhibited by 60% ($P < 0.05$) the growth of MCF-7ras tumours after 7 weeks of treatment. No apparent toxicity was reported during mice treatment as their weight

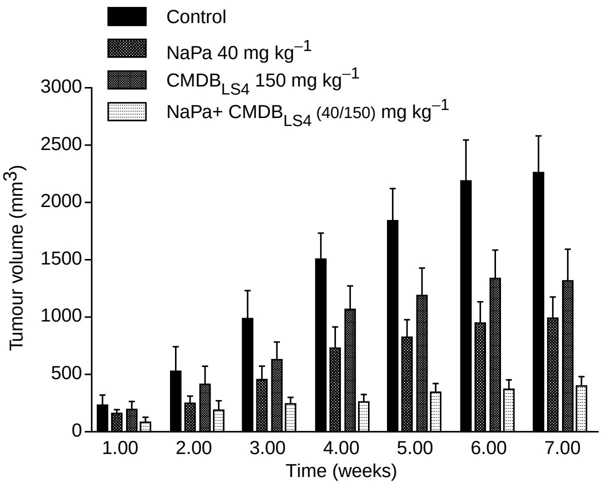

**Figure 6** In vivo growth inhibition of MCF-7ras carcinoma by NaPa, $CMDB_{LS4}$ and $CMDB_{LS4}$-NaPa-combination ($CMDB_{LS4}$/NaPa ratio = 3.75). MCF-7ras cells were inoculated in nude mice as described in 'Materials and Methods'. After 4 weeks, mice were treated with NaPa (40 mg $kg^{-1}$), $CMDB_{LS4}$ (150 mg $kg^{-1}$) and $CMDB_{LS4}$ combined with NaPa (150 mg $kg^{-1}$ and 40 mg $kg^{-1}$ respectively) twice a week for 7 weeks. Each chart represents a mean tumour volume ($mm^3$) (± SD; $n$ = 10)

remained stable between day 0: 28.4 g ± 1 and day 7: 30 ± 1. $CMDB_{LS4}$ (150 mg $kg^{-1}$ twice a week) inhibited the tumour growth by 40% ($P < 0.05$) after 7 weeks of treatment without toxicity. When NaPa and $CMDB_{LS4}$ were administred at the same dose but in combination, the tumour growth was inhibited by 83% ($P \leq 0.001$) after 7 weeks and no mice toxicity (mice weight at day 0: 28.9 g ± 0.6, day 7: 33.0 ± 0.01) was noticed.

### DISCUSSION

Our study demonstrates, for the first time to our knowledge, that NaPa enhances dextran derivative, $CMDB_{LS4}$, antiproliferative effect on breast cancer MCF-7ras cells in vitro and in vivo. Indeed, NaPa or $CMDB_{LS4}$, delivered alone for 7 weeks, inhibited MCF-7ras tumour growth by 60% and 40%, respectively, while the $CMDB_{LS4}$-NaPa combination decreased MCF-7ras tumour growth by 83% without any toxicity. The effectiveness of the NaPa and $CMDB_{LS4}$ combination could be explained by their distinct mechanisms of action. MCF-7ras breast cancer cells secrete an important amount of mitogenic growth factors such as TGFβ and PDGF (Bronzert et al, 1987; Dickson et al, 1987; Knabbe et al, 1987). The mitogenic effects of these growth factors could be reduced by inhibition of their synthesis or/and their action on target cells. In our study, we showed that a treatment of cells with NaPa decreased the mitogenic activity of MCF-7ras conditioned medium on BALBc/3T3. This could be explained by a modulation of the synthesis and the release of growth factors like TGFβ in MCF-7ras breast cancer cells as reported previously by our laboratory (Thibout et al, 1998). Concerning $CMDB_{LS4}$, we have demonstrated here that when added to CM it inhibited CM mitogenic effect on BALBc/3T3 fibroblasts. This finding argues for $CMDB_{LS4}$ interactions with growth factors contained in CM. Indeed, previous studies showed that dextran derivative interacted with heparin-binding growth factors like TGFβ, PDGFBB or FGF-2 and inhibited their mitogenic effect (Bagheri-Yarmand et al, 1998a, b). All our and others' observations suggest that NaPa

© 2001 Cancer Research Campaign and $CMDB_{LS4}$ act on distinct targets involved in the tumour development. NaPa alters the mitogenic growth factor production and rendered the tumour cells quiescent in the G1 phase while $CMDB_{LS4}$ interacts with MCF-7ras growth factors and inhibits their mitogenic activities.

The additive and synergistic effect in vitro and in vivo was observed when the concentrations of $CMDB_{LS4}$ were 1 to 8-fold higher than NaPa concentration. If the $CMDB_{LS4}$/NaPa ratio in vitro is lower than 0.1, the concentrations of $CMDB_{LS4}$ are probably too low to potentiate the NaPa inhibitory effect although they are efficient when $CMDB_{LS4}$ is used alone. When the ratio of $CMDB_{LS4}$ and NaPa concentrations is higher than 5, the inhibitory effect was unchanged suggesting that maximal efficiency of treatment is achieved at ratio 5. Within this same ratio, the combined antiproliferative effect was synergistic when NaPa and $CMDB_{LS4}$ concentrations were low and it became additive in the case of higher concentrations. This complexity of interaction between 2 drugs may be due to the fact that at different concentrations the drugs could induce various effects. For example, we showed that NaPa at concentration 5 mM, begun to induce G1–S transition arrest, whereas at higher concentration (40 mM), it generated the apoptosis after 48 h of treatment of 10% of cell as revealed by a single annexin V labelling and after 72 h, the destruction of cell membrane integrity probably due to necrosis or/and later apoptosis as showed by annexin V/PI labelling. The difficulty to distinguish the apoptotic cells from the necrotic ones may be, in part, explained by the fact that some drugs at high doses can induce new morphological features characteristic of both apoptosis and necrosis chimerically termed aponecrosis as proposed by Formigli et al (2000). MCF-7ras cell apoptosis induced by NaPa was previously described and associated with a reduction of the antiapoptotic *Bc12* gene expression (Adam et al, 1995).

It is noteworthy that NaPa at high concentrations can induce pathological effects (Thibault et al, 1994; Chang et al, 1999) that may involve an induction of cell death as we observed in vitro. The use of combined treatments, reported in this paper, should reduce the dose of NaPa without decreasing a therapeutic efficacy and consequently should limit side effects. Indeed, we observed here an important MCF-7ras xenograft growth cytostatic inhibition (83%) using much lower concentrations of NaPa which are not toxic. Moreover, NaPa does not induce a multi-drug resistance (Adam et al, 1995).

In conclusion, our results clearly indicate the complementary action of NaPa and $CMDB_{LS4}$ on the autocrine or/and paracrine loops of growth factors to induce, at low doses, a tumour cell cytostatic effect. An important perspective of this study is based on the improvement of the antiproliferative properties of dextran derivatives depending on active subunit substitutions (Bagheri-Yarmand et al, 1992). Currently, we are investigating the antiproliferative effect of a carboxymethybenzylamide dextran derivative substituted with active phenylacetate subunits.

### ACKNOWLEDGEMENTS

The authors thank L Dahri-Correia and J Correia (LS4 sample) and P Bissière for technical advices. We thank Dr G Palmieri and Dr Frojmock for language correction. This work was supported by a grant from the 'Association pour la Recherche sur le Cancer', 'Ligue contre le Cancer', 'Ministère de l'Education Nationale' and Sterilyo Laboratories.

### REFERENCES

Adam L, Crepin M, Savin C and Israel L (1995) Sodium phenylacetate induces growth inhibition and Bc12 down regulation and apoptosis in MCF-7ras cells in vitro and in nude mice. *Cancer Res* **55**: 5156–5160

Adam L, Crepin M and Israel L (1997) Tumor growth inhibition, apoptosis, and Bcl-2 down-regulation of MCF-7ras by sodium phenylacetate and tamoxifene combination. *Cancer Res* **57**: 1023–1029

Albini A, Graf J, Kitten GT, Kleinlan HK, Martin GR, Veillette A and Lippman ME (1986) 17 β estradiol regulates and v-Ha-ras transfection constitutively enhances MCF7 breast cancer cell interactions with basement membrane. *Proc Natl Acad Sci USA* **83**: 8182–8186

Bagheri-Yarmand R, Morere JF, Letouneur D, Jozefonvicz J, Israel L and Crepin M (1992) Inhibitory effect of dextran derivatives in vitro on the growth characteristics of premalignant and malignant human mammary epithelial cell lines. *Anticancer Res* **12**: 1641–1646

Bagheri-Yarmand R, Liu JF, Ledoux D, Morere JF and Crépin M (1997) Inhibition of human breast epithelial HBL 100 cell proliferation by a dextran derivative (CMDB7) with FGF2 autocrine loop. *Biochem Biophys Res Commun* **239**: 424–428

Bagheri-Yarmand R, Kourbali Y, Mabilat C, Morere JF, Martin A, Lu LH, Soria C, Jozefonvicz J and Crepin M (1998a) The supression of fibroblast growth factor 2/fibroblast growth factor 4-dependent tumor angiogenesis and growth by the anti-growth factor activity of dextran derivative (CMDB7). *Br J Cancer* **78**: 111–118

Bagheri-Yarmand R, Kourbali Y, Morere JF, Jozefonvicz J and Crepin M (1998b) Inhibition of MCF-7ras tumor growth by benzylamide dextran: blockage of the paracrine effect and receptor binding of transforming growth factor β1 and platelet-derived growth factor-BB. *Cell Growth Differ* **9**: 497–504

Bagheri-Yarmand R, Kourbali Y, Rath AM, Vassy R, Martin A, Jozefonvicz J, Soria C, He L and Crépin M (1999) Carboxymethyl benzylamide dextran blocks angiogenesis of MDA-MB 435 breast carcinoma xenografted in fad pad and its lung metastases in nude mice. *Cancer Res* **59**: 507–510

Berenbaum KC (1981) Criteria for analysing interaction between biologically active agents. *Adv Cancer Res* **25**: 269–335

Bronzert K, Pantazis P, Antoniades HN, Kasid A, Davidson N, Dickson RB and Lippman ME (1987) Synthesis and secretion of platelet-derived growth factor by human breast cancer cell lines. *Proc Natl Acad Sci USA* **84**: 5763–5767

Chang SM, Kuhn JG, Robin HI, Clifford Schold S, Spence AM, Berger MS, Metha MP, Bozik ME, Pollack I, Schiff D, Gilbert M, Rankin C and Prados MD (1999) Phase II study of phenylacetate in patients with recurrent malignant glioma: a north American brain tumor concortium. *J Clin Oncol* **17**: 984–990

Chaubet F, Champion J, Maïga O, Mauray S and Jozefonvicz J (1995) Synthesis and structure-anticoagulant property relationships of functionalized dextrans: CMDBS. *Carbohydrate Polymers* **28**: 145–152

Dickson RB, Kasid A, Huff KK, Bates SE, Knabbe C, Bronzert D, Gelman EP and Lippman ME (1987) Activation of growth factor secretion in tumorigenic states of breast cancer induced by 17β estradiol or V-Ha-ras oncogene. *Proc Natl Acad Sci USA* **84**: 837–841

Ferrandina G, Melichar B, Loercher A, Verschaegen CF, Kudelka AP, Edwards CL, Scambia G, Kavanagh JJ, Abbruzzese JL and Freedman RS (1997) Growth inhibitory effects of sodium phenylacetate (NSC 3039) on ovarian carcinoma cells in vitro. *Cancer Res* **57**: 4309–4315

Formigli L, Papucci L, Tani A, Schiavone N, Tempestini A, Orlandini GE, Capaccioli S and Orlandini SZ (2000) Aponecrosis: morphological and biochemical exploration of a syncretic process of cell death sharing apoptosis and nerosis. *J Cell Physiol* **182**: 41–49

Knabbe C, Wakefield L, Flanders K, Kasid A, Derynck R, Lippman ME and Dickson RB (1987) Evidence that TGF β is a hormonally regulated negative growth factor in human breast cancer cell lines. *Cell* **48**: 417–428

Mauzac M and Jozefonvicz J (1984) Anticoagulant activity of dextran derivatives. Part I: Synthesis and characterization. *Biomaterials* **5**: 301–304

Mosmann T (1983) Rapid colorimetric assay for cell growth and survival: Application to proliferation and cytotoxicity assays. *J Immunol Methods* **65**: 55–63

Prasanna P, Thilbault A, Liu L and Samid D (1996) Lipid metabolism as a target for brain cancer therapy: synergistic activity of lovastatin and sodium phenylacetate and phenylbutyrate. *Clin Cancer Res* **2**: 865–872

Samid D, Shack S and Myers CE (1993) Selective growth arrest and phenotypic reversion of prostate cancer cells in vitro by non toxic pharmalogical concentration of phenylacetate. *J Clin Invest* **91**: 2288–2295

Samid D, Ram Z, Hudgins WR, Shack S, Liu L, Walbindge S, Oldfild EH and Myers CE (1994) Selective activity of phenylacetate against malignant

 *© 2001 Cancer Research Campaign*

gliomas: ressemblance to fetal brain damage in phenylketonuria. *Cancer Res* **54**: 891–895

Samid D, Hudgins WR, Shack S, Liu L, Prasanna P and Myers CE (1997) Phenylacetate and phenylbutyrate as novel, nontoxic differenciation inducers. *Adv Exp Med Biol* **400A**: 501–505

Samid D, Wells M, Greene ME, Shen W, Palmer CN and Thibault A (2000) Peroxisome proliferator-activated receptor gamma as a novel target in cancer therapy: binding and activation by an aromatic fatty acid with clinical antitumor activity. *Clin Cancer Res* **3**: 933–941

Sommers CL, Papageorge A, Wilding G and Gelmann EP (1990) Growth properties and tumorigenesis of MCF-7 cells transfected with isogenic mutants of ras$^{H}$. *Cancer Res* **50**: 67–71

Spandidos DA and Agnantis NJ (1984) Human malignant tumors of the breast as compared to their respective normal tissue have elevated expression of the Harvey ras oncogene: *Anticancer Res* **4**: 269–272

Thibault A, Cooper MR, Figureg WD, Venzan DJ, Sartor OA, Tompkins AC, Weinberg MS, Headlee DJ, Mc Coll Na, Samid D and Myers CE (1994) A phase I and pharmacokinetic study of intravenous phenylacetate in patients with cancer. *Cancer Res* **54**: 1690–1694

Thibout D, Di Benedetto M, Kraemer M, Sainte-Catherine O, Derbin C and Crepin M (1998) Sodium phenylacetate modulates the synthesis of autocrine and paracrine growth factors secreted by breast cancer cell lines. *Anticancer Res* **18**: 2657–2662

© 2001 Cancer Research Campaign
